# Supplementary material for: Temperature-adaptive hydrogel optical waveguide with soft tissue-affinity for thermal regulated interventional photomedicine
Source: Nat Commun. 2022 Dec 16;13:7789. doi: 10.1038/s41467-022-35440-w (PMC9758120; doi:10.1038/s41467-022-35440-w)
Supplement: Supplementary file 1 — Supplementary Information [file 41467_2022_35440_MOESM1_ESM.pdf]

Supplementary Information

Temperature-Adaptive Hydrogel Optical Waveguide with Soft Tissue-Affinity for Thermal Regulated Interventional Photomedicine

Guoyin Chen[1], Kai Hou[1,], Nuo Yu[1], Peiling Wei[1], Tao Chen[1], Caihong Zhang[1], Shun Wang[1], Hongmei Liu[1], Ran Cao[1,]*, Liping Zhu[1], Benjamin S. Hsiao[1], Meifang Zhu[1,]**

[1] State Key Laboratory for Modification of Chemical Fibers and Polymer Materials, College of Materials Science and Engineering, Donghua University, 2999 North Renmin Road, Shanghai 201620, China

[2] Department of Chemistry, Stony Brook University, Stony Brook, NewYork, 11794, USA

* To whom correspondence be addressed. E-mail: R. Cao (rancao@dhu.edu.cn), K. Hou (houkai711@dhu.edu.cn), M.F. Zhu (zmf@dhu.edu.cn)

Supplementary Table 1. Detailed pre-gel solutions/hydrogels of thermosensitive hydrogel.

Samples	NIPAM (g)	DMAAm (g)	PEGDA (g)	I2959 (g)	Deionized Water (g)
$(N_{100}D_0)_{50}$	15.0	0.0	0.15	0.075	15.0
$(N_{90}D_{10})_{50}$	13.5	1.5	0.15	0.075	15.0
$(N_{80}D_{20})_{50}$	12.0	3.0	0.15	0.075	15.0
$(N_{70}D_{30})_{50}$	10.5	4.5	0.15	0.075	15.0
$(N_{60}D_{40})_{50}$	9.0	6.0	0.15	0.075	15.0
$(N_{50}D_{50})_{50}$	7.5	7.5	0.15	0.075	15.0

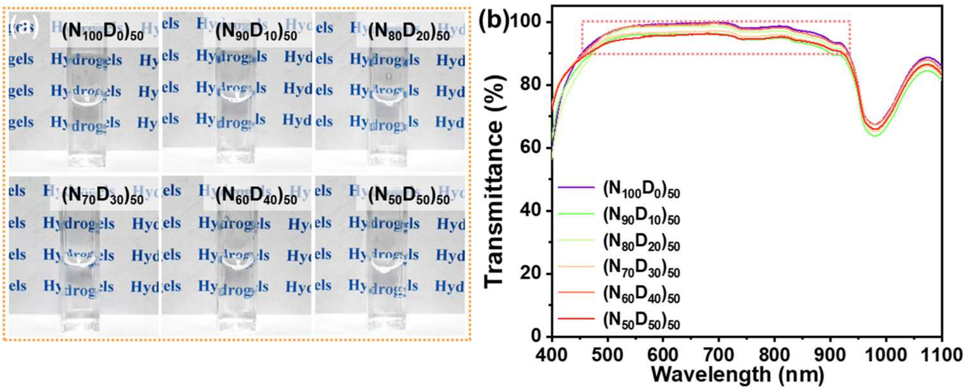

Supplementary Figure 1. Optical properties of the $(N_XD_{100-X})_{50}$ hydrogels: (a) Photographs and (b) transmittance of $(N_XD_{100-X})_{50}$ hydrogels with different DMAAm contents.

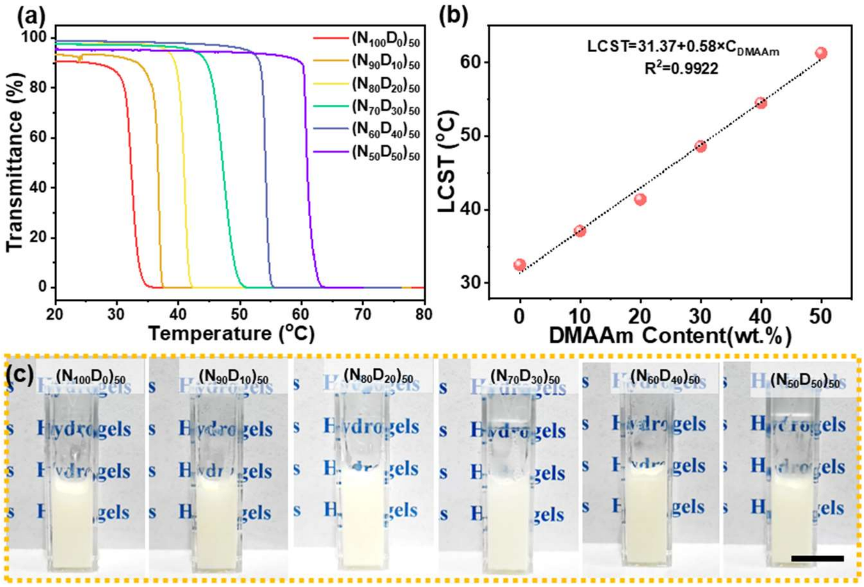

Supplementary Figure 2. Thermal-sensitivity of the $(N_XD_{100-X})_{50}$ hydrogels dependent on the DMAAm content: (a) Temperature dependent transmittance of p(NIPAm-*co*-DMAAm) hydrogels with different DMAAm contents; (b) LCST of p(NIPAm-*co*-DMAAm) hydrogels dependent on the DMAAm content, (λ = 515 nm); (c) Photographs of $(N_XD_{100-X})_{50}$ hydrogels with different DMAAm contents under the temperature > LCST.

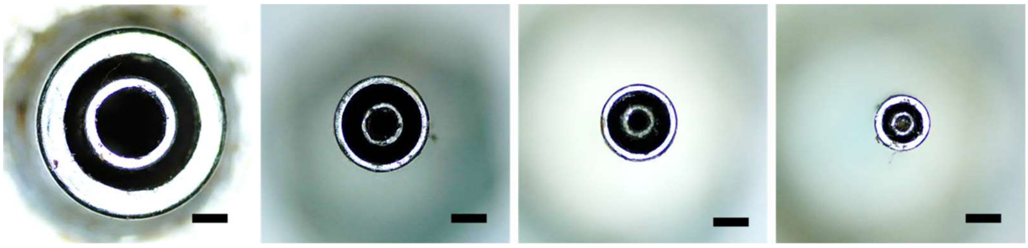

Supplementary Figure 3. Cross-sectional morphologies of the coaxial needle used for THFOW fabrication, scale bar = 500 μm.

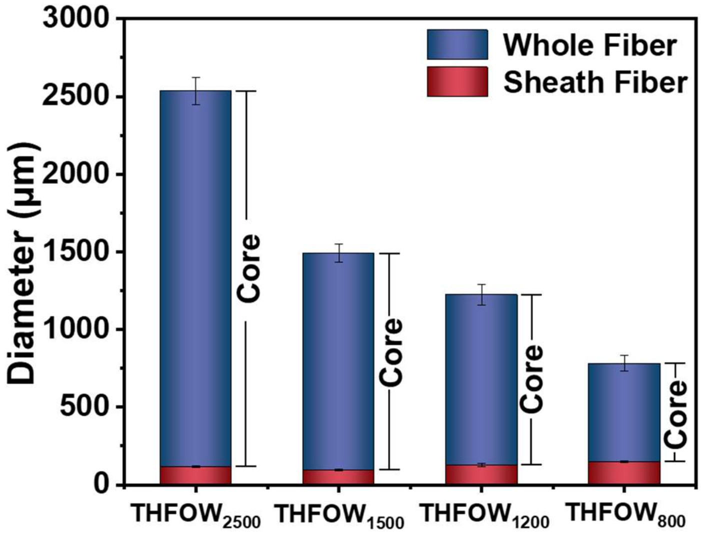

Supplementary Figure 4. Diameters of the fabricated THFOWs (n = 5 independent experiments), data were presented as mean ± SD.

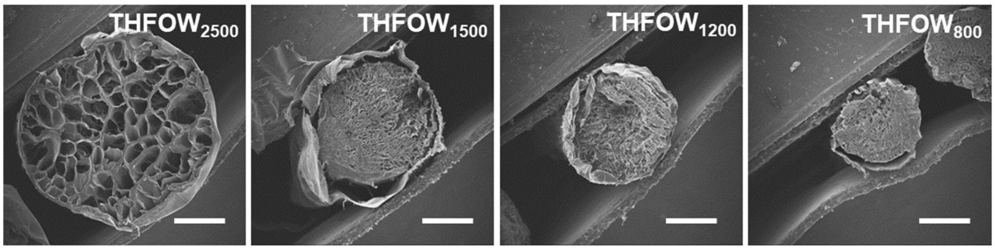

Supplementary Figure 5. SEM images of the fabricated THFOWs, scale bar is 500 μm. A representative image of three independent samples from each group is shown in this Figure.

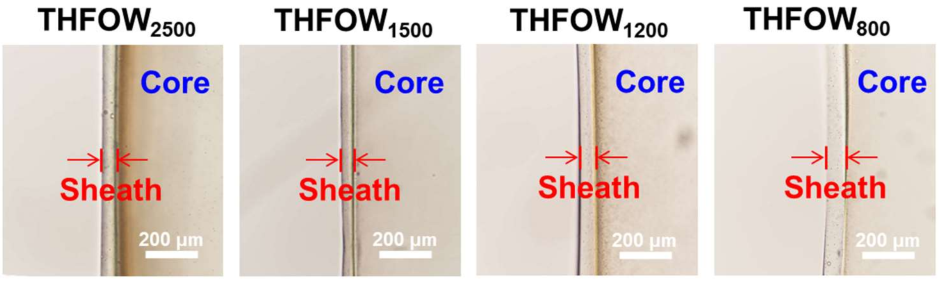

Supplementary Figure 6. Profile photos of the fabricated THFOWs under 10×20 lens.

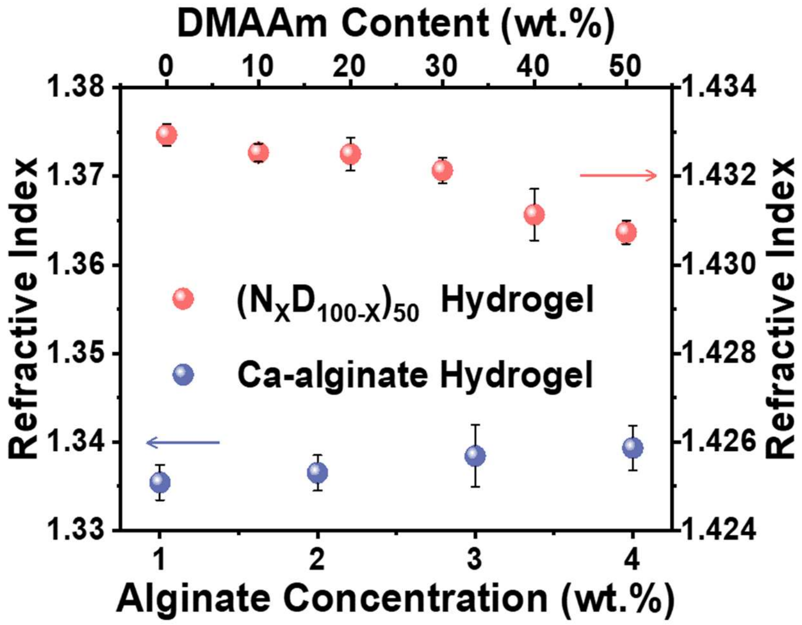

Supplementary Figure 7. Refractive index of the core and sheath raw-materials (n = 3 independent experiments), data were presented as mean ± SD.

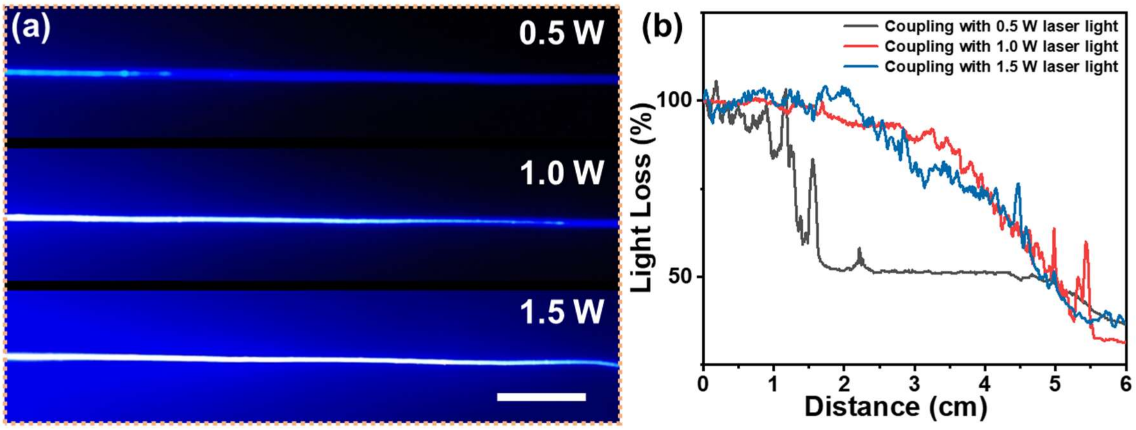

Supplementary Figure 8. **Light propagation through the THFOW by coupling with 450 nm laser light:** (a) Light transmission of $THFOW_{800}$ coupling with different laser intensities, scale bar is 1 cm; (b) Light loss through the $THFOW_{800}$ that coupling with different laser intensities.

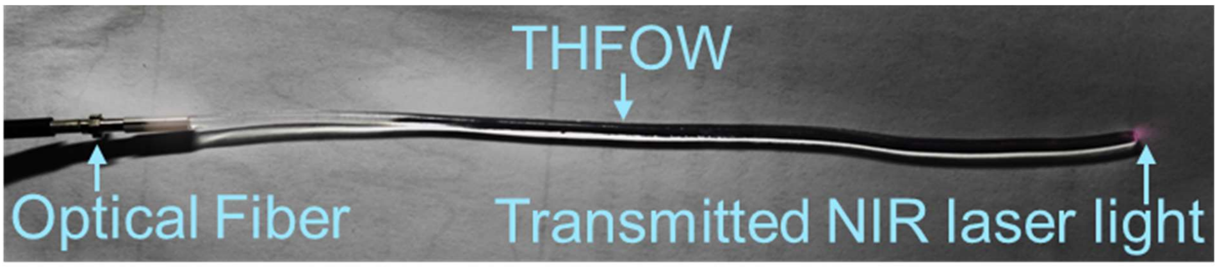

Supplementary Figure 9. NIR light (915 nm) propagated through a $THFOW_{2500}$.

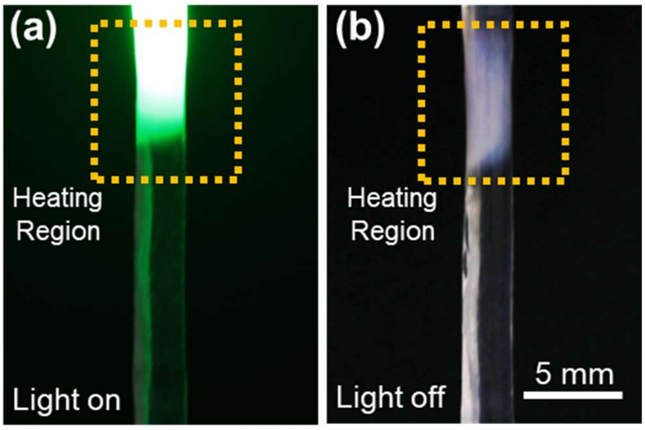

Supplementary Figure 10. Photos of heating region with light on (a) and light off (b).

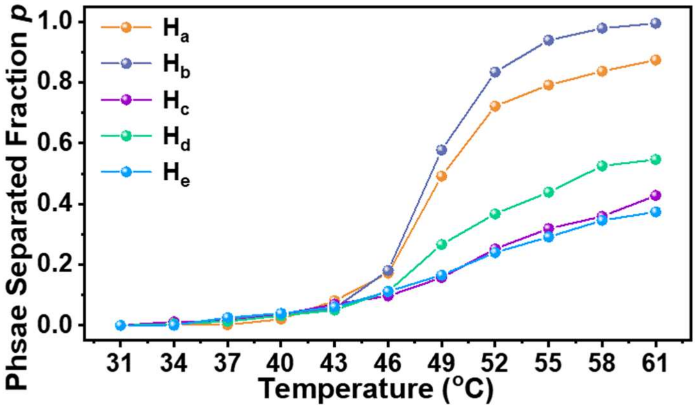

Supplementary Figure 11. Temperature dependency of phase separated fraction p for different proton types of $(N_{70}D_{30})_{50}$ hydrogel.

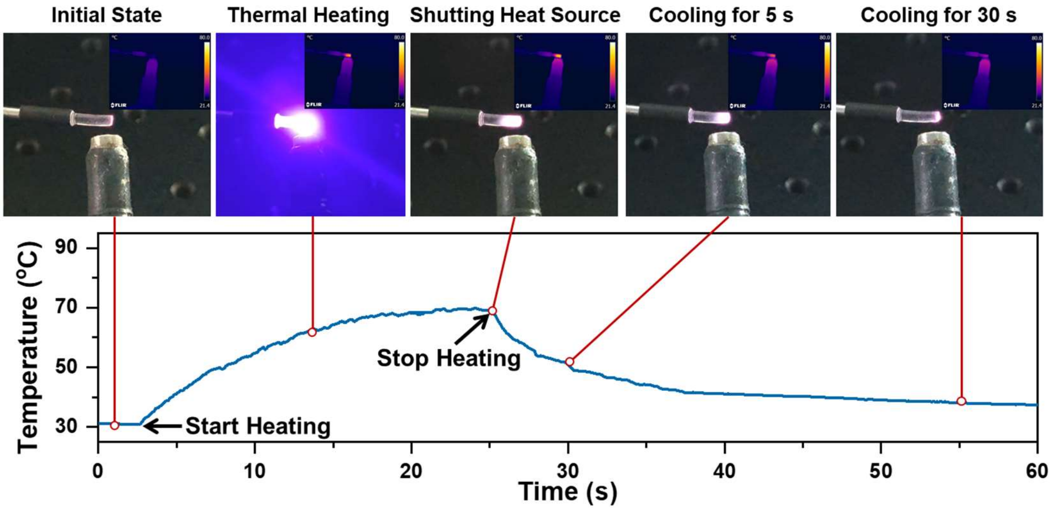

Supplementary Figure 12. State variation of THFOW before/after phase-separation (coupling with 915 nm NIR light).

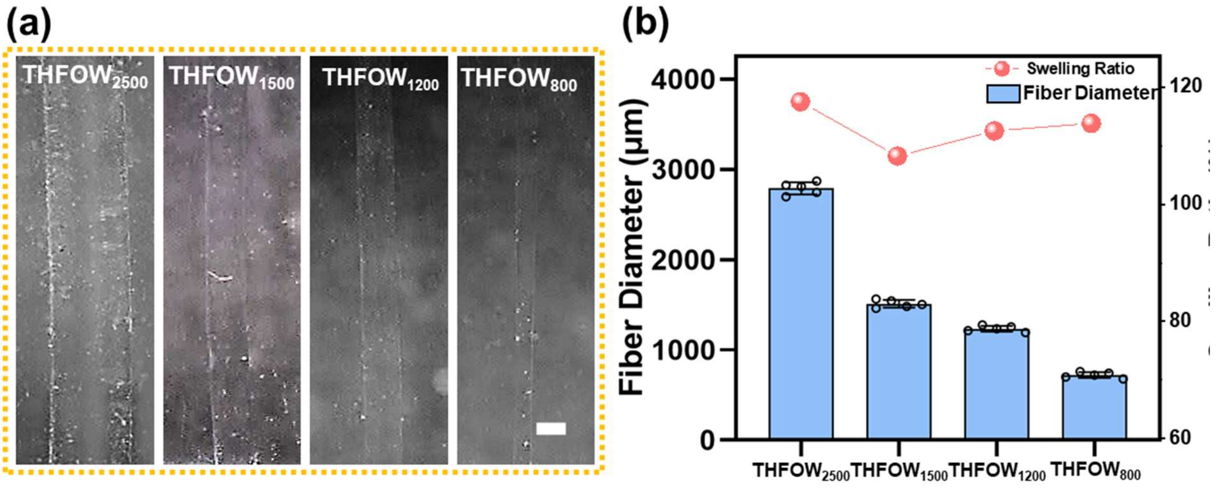

Supplementary Figure 13. Diameters analysis: (a) Optical micrographs of the THFOWs after removing the sheath Ca-alginate hydrogel layer and selling to the equivalent state, scale bar = 1000 μm; (b) Relavant fiber diameters of the THFOWs (n = 5 independent experiments), data were presented as mean ± SD.

THFOW with different size showed a different swelling behaviour, the swelling ratio was increased in the order of $THFOW_{2500}$, $THFOW_{800}$, $THFOW_{1200}$ and $THFOW_{1500}$, which was corresponding with the size of spinning needle for different THFOW (Figure S3). A large,which the swelling ratio was almost maintain at the 10% compare to the THFOW fabricated after inmersing for 7 days. Which was due to the ralative high crosslinking density and the sheath Ca-alginate fiber.

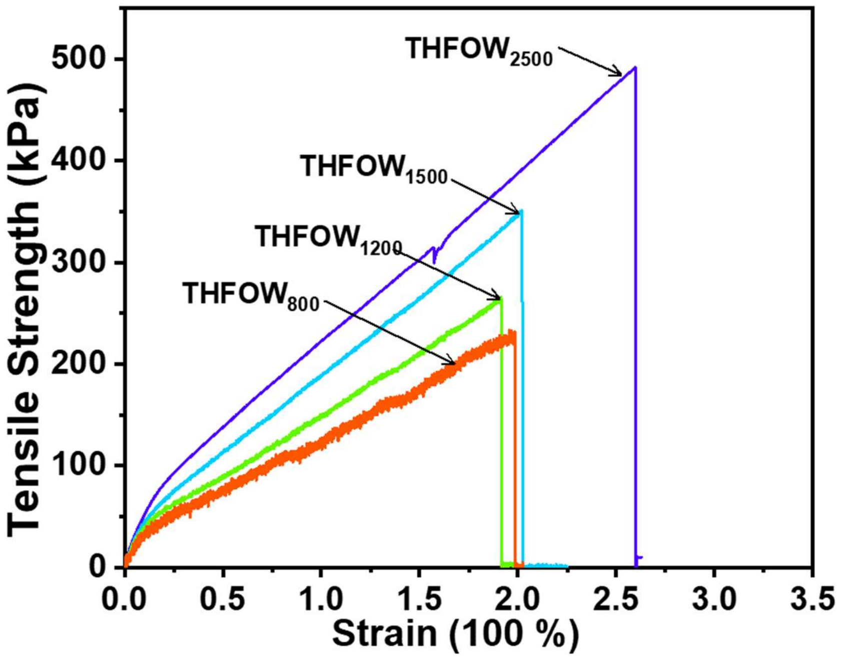

Supplementary Figure 14. Strain-Strength curves of ther fabricated THFOW.

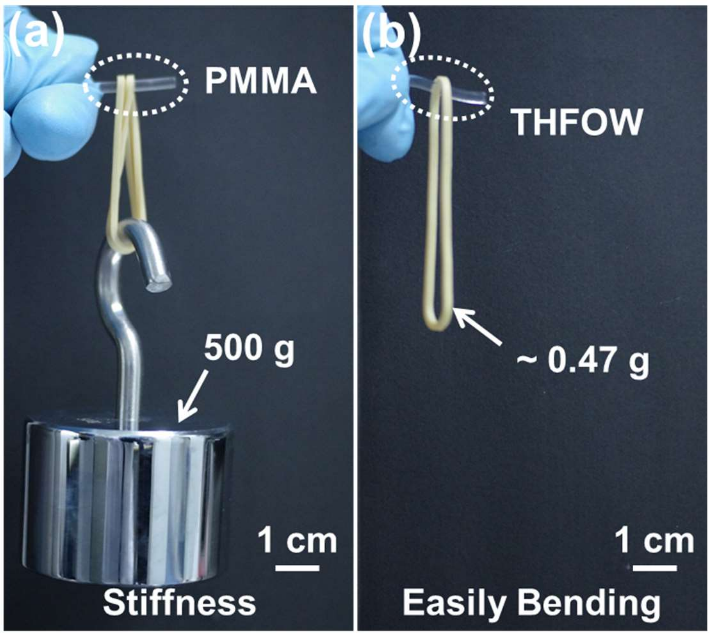

Supplementary Figure 15. Visual comparison of modulus between PMMA Fiber (a) and the fabricated THFOW (b).

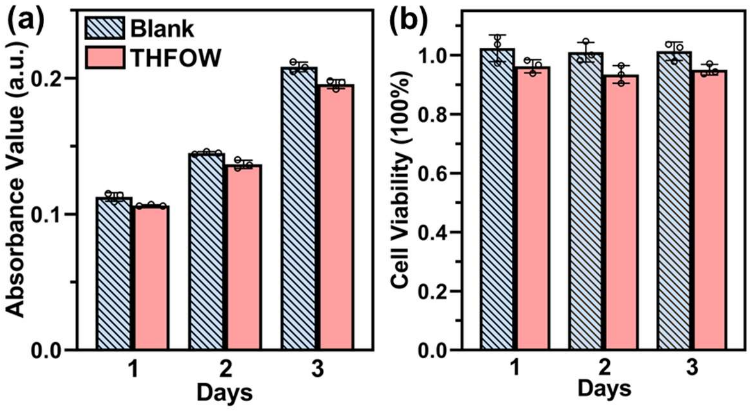

Supplementary Figure 16. Cytotoxicity of the THFOW: (a) Relative proliferation and (b) Live/dead assay of Hela cells on THFOWs compared to blank at 1, 2 and 3 days (n = 3 biologically independent samples), data were presented as mean ± SD.

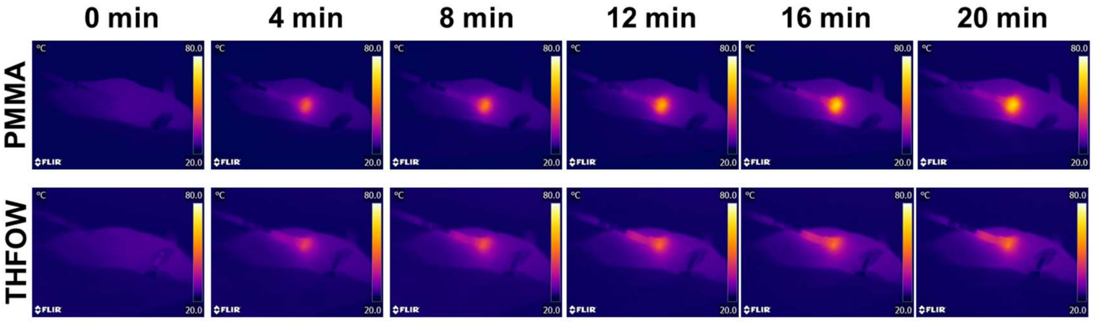

Supplementary Figure 17. Infrared thermal images of the mice in PMMA and THFOW group during photothermal therapy.

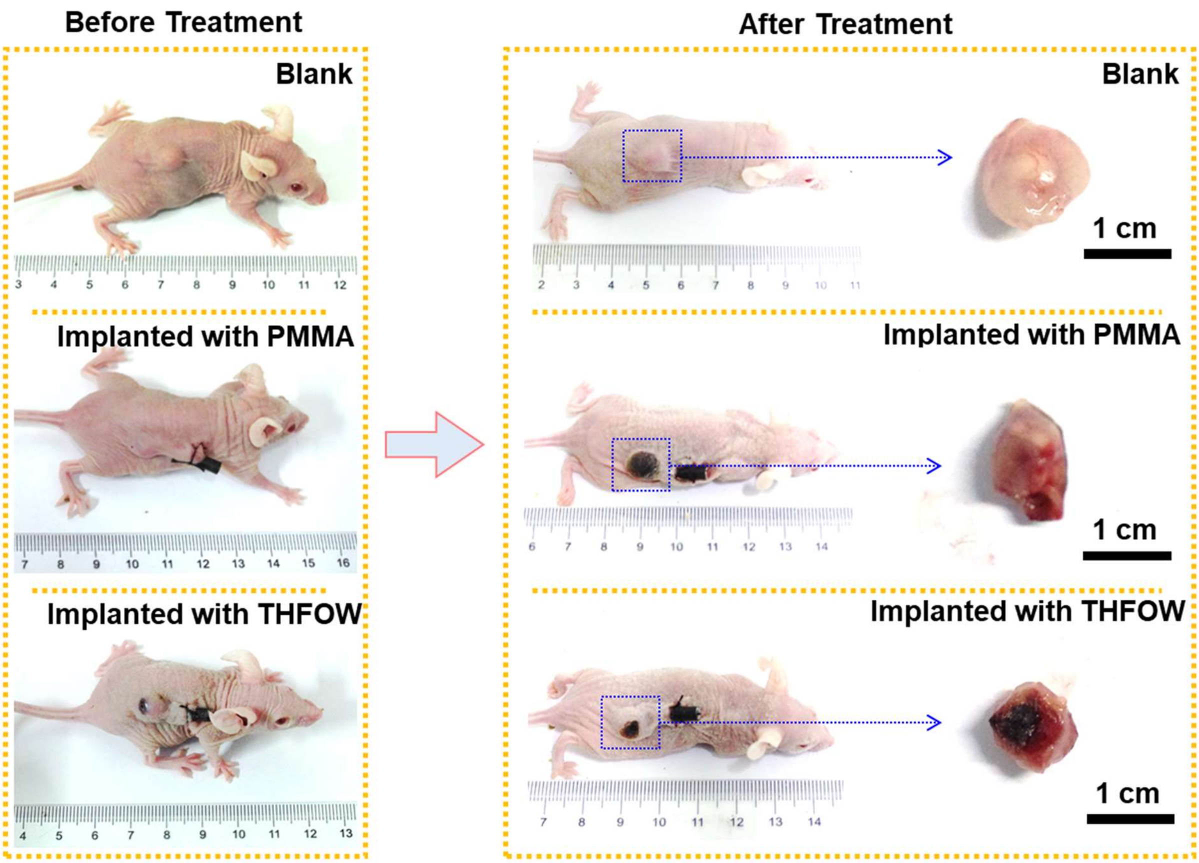

Supplementary Figure 18. Photos of mice and the tumor before and after cancer therapy.

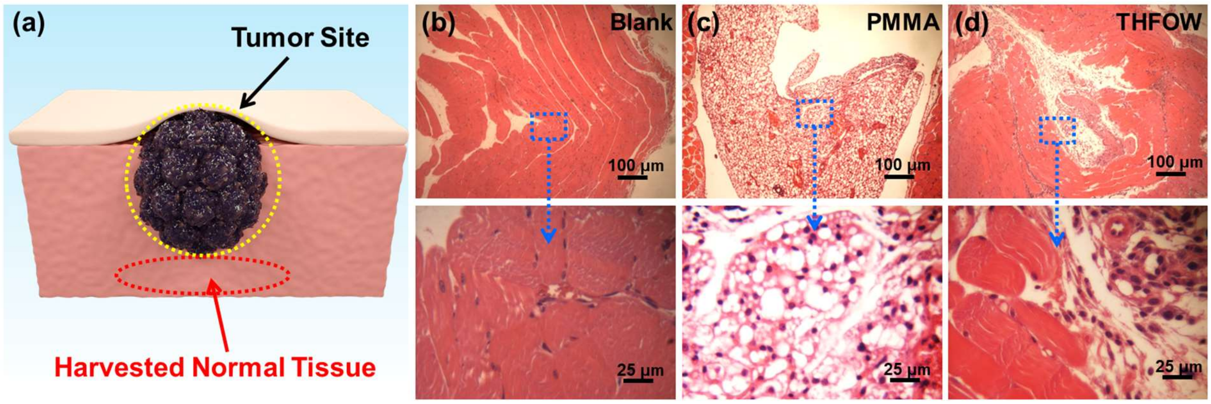

Supplementary Figure 19. **Clinicopathologic analysis of the tissues under the tumor site**: (a) Schematic illustrate of the harvested normal tissue that for clinicopathologic analysis; (b-d) H&E stained tissues slices harvested from the tissues of mice under the tumor site for different groups. A representative image of three independent samples from each group is shown in (b-d).

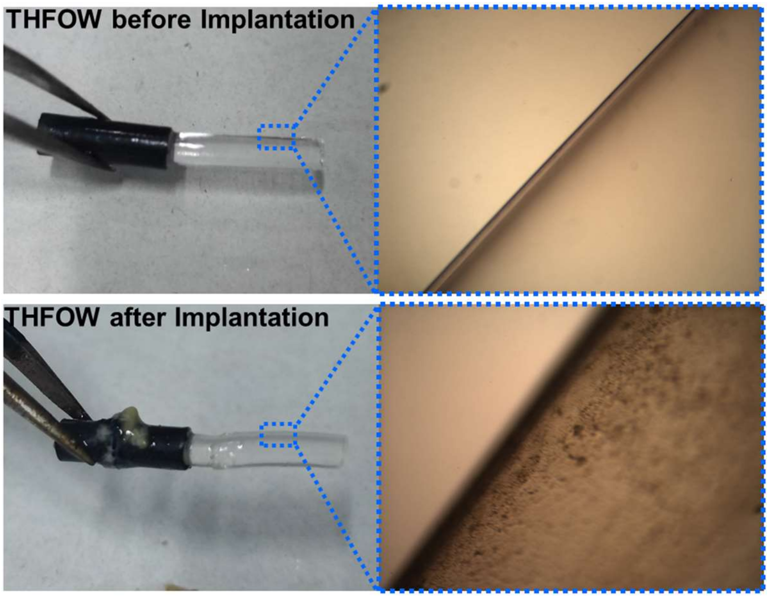

Supplementary Figure 20. THFOW before/after implanted into the mouse for photothermal therapy.
