# Supplementary material for: Temperature-adaptive hydrogel optical waveguide with soft tissue-affinity for thermal regulated interventional photomedicine
Source: Nat Commun. 2022 Dec 16;13:7789. doi: 10.1038/s41467-022-35440-w (PMC9758120; doi:10.1038/s41467-022-35440-w)
Supplement: Supplementary file 3 — Reporting Summary [file 41467_2022_35440_MOESM3_ESM.pdf]

nature portfolio

Corresponding author(s): Kai Hou, Ran Cao, Meifang Zhu

Last updated by author(s): Feb 20, 2022

Reporting Summary

Nature Portfolio wishes to improve the reproducibility of the work that we publish. This form provides structure for consistency and transparency in reporting. For further information on Nature Portfolio policies, see our Editorial Policies and the Editorial Policy Checklist.

Statistics

For all statistical analyses, confirm that the following items are present in the figure legend, table legend, main text, or Methods section.

n/a	Confirmed	
☐	☒	The exact sample size (*n*) for each experimental group/condition, given as a discrete number and unit of measurement
☐	☒	A statement on whether measurements were taken from distinct samples or whether the same sample was measured repeatedly
☒	☐	The statistical test(s) used AND whether they are one- or two-sided<br>*Only common tests should be described solely by name; describe more complex techniques in the Methods section.*
☐	☒	A description of all covariates tested
☐	☒	A description of any assumptions or corrections, such as tests of normality and adjustment for multiple comparisons
☐	☒	A full description of the statistical parameters including central tendency (e.g. means) or other basic estimates (e.g. regression coefficient) AND variation (e.g. standard deviation) or associated estimates of uncertainty (e.g. confidence intervals)
☒	☐	For null hypothesis testing, the test statistic (e.g. *F*, *t*, *r*) with confidence intervals, effect sizes, degrees of freedom and *P* value noted<br>*Give P values as exact values whenever suitable.*
☒	☐	For Bayesian analysis, information on the choice of priors and Markov chain Monte Carlo settings
☒	☐	For hierarchical and complex designs, identification of the appropriate level for tests and full reporting of outcomes
☒	☐	Estimates of effect sizes (e.g. Cohen's *d*, Pearson's *r*), indicating how they were calculated

Our web collection on statistics for biologists contains articles on many of the points above.

Software and code

Policy information about availability of computer code

Data collection	FE-SEM, NMR, UV-vis, FTIR, Infrared Camera, stereoscopic microscopy, fluorescence microscope, refractometer, handheld optical power meter, Instron 5969 universal test machine, microplate reader
Data analysis	All the statistical analyses were carried out by Origin 8.5, Excel 2016 and GraphPad Prism 6.

For manuscripts utilizing custom algorithms or software that are central to the research but not yet described in published literature, software must be made available to editors and reviewers. We strongly encourage code deposition in a community repository (e.g. GitHub). See the Nature Portfolio guidelines for submitting code & software for further information.

Data

Policy information about availability of data

All manuscripts must include a data availability statement. This statement should provide the following information, where applicable:

- Accession codes, unique identifiers, or web links for publicly available datasets
- A description of any restrictions on data availability
- For clinical datasets or third party data, please ensure that the statement adheres to our policy

All the data supporting the work are available within the main text of this article and its Supplementary Information. The raw data of Figures 3 – 6, and Supplementary Figures 1, 2, 4, 7, 11 – 14, 16 can be found in the Source Data. Source data are provided with this paper.

Field-specific reporting

Please select the one below that is the best fit for your research. If you are not sure, read the appropriate sections before making your selection.

☒ Life sciences ☐ Behavioural & social sciences ☐ Ecological, evolutionary & environmental sciences

For a reference copy of the document with all sections, see nature.com/documents/nr-reporting-summary-flat.pdf

Life sciences study design

All studies must disclose on these points even when the disclosure is negative.

Sample size	Sample size was determined according to previous experimental experiences and/or well established reported works (Adv. Mater. 2016, 28, 245–253; Natl. Sci. Rev., 2021, 8(6), nwaa156;Nano Lett., 2018, 18(9): 6037-6044.). No statistical method was used to predetermine sample size. The in vivo sample size was five animals per treatment group. And at least three biological replicates were considered for most the in vitro studies.
Data exclusions	No data was exluded
Replication	Experiments were repeated and experimental finding were reproducible. All experiments were run at least in triplicate
Randomization	All experimental samples and mice were allocated randomly to each group.
Blinding	Mice were used this work. Binding and randomization were applied to all experiments

Reporting for specific materials, systems and methods

We require information from authors about some types of materials, experimental systems and methods used in many studies. Here, indicate whether each material, system or method listed is relevant to your study. If you are not sure if a list item applies to your research, read the appropriate section before selecting a response.

Materials & experimental systems

n/a	Involved in the study
☒	☐ Antibodies
☐	☒ Eukaryotic cell lines
☒	☐ Palaeontology and archaeology
☐	☒ Animals and other organisms
☒	☐ Human research participants
☒	☐ Clinical data
☒	☐ Dual use research of concern

Methods

n/a	Involved in the study
☒	☐ ChIP-seq
☒	☐ Flow cytometry
☒	☐ MRI-based neuroimaging

Eukaryotic cell lines

Policy information about cell lines

Cell line source(s)	Hela cells were obtained from American type culture collection
Authentication	The cell line we used was morphologically confirmed according to the information provide by American type culture collection
Mycoplasma contamination	All cell lines were tested for mycoplasma contamination. No mycoplasma contamination was found.
Commonly misidentified lines (See ICLAC register)	No commonly misidentified cell lines was used

Animals and other organisms

Policy information about studies involving animals; ARRIVE guidelines recommended for reporting animal research

Laboratory animals	Five-week-old BALB/c nu/nu mice (19±2 g) were used as animal model in this work; Mice were housed in groups of 5 mice per individually cage in a 12-h light-dark cycle (8:00 – 20:00 light; 20:00 – 8:00 dark), where the environment is kept at constant room temperature (21 ± 1 °C) and relative humidity of 40 – 70%.
Wild animals	The study did not involve wild animals.

Field-collected samples	The study did not involves samples collected from field.
Ethics oversight	All animal experiments were carried out under the permission by the Ethical Committee for Animal Care of Donghua University.

Note that full information on the approval of the study protocol must also be provided in the manuscript.
